# Bridging the gap: decomposing sources of gender yield gaps in Uganda groundnut production

**DOI:** 10.1080/09718524.2019.1621597

**Published:** 2019-06-12

**Authors:** Johnny Mugisha, Christopher Sebatta, Kai Mausch, Elizabeth Ahikiriza, David Kalule Okello, Esther M. Njuguna

**Affiliations:** a Department of Agribusiness and Natural Resource Economics, Makerere University, Kampala, Uganda;; b World Agroforestry (ICRAF), Nairobi, Kenya;; c NARO/National Semi-Arid Resources Research Institute (NaSARRI), Soroti, Uganda;; d International Crops Research Institute for the Semi-Arid Tropics (ICRISAT), Nairobi, Kenya

**Keywords:** Blinder-Oaxaca model, female-managed plot, male-managed plot, gender yield gap

## Abstract

Female plot managers in Sub-Saharan Africa often realize significantly lower crop yields than their male counterparts. Even for legumes, which are often referred to as ‘women’s crops’, yields are significantly lower. This study investigated the underlying causes of this gender yield gap in groundnut production. The analysis is based on survey data from 228 farm households from two groundnut growing regions in Uganda. We used the Blinder-Oaxaca model to decompose factors that contribute to this yield gap. Results show 63% and 44% gender yield gaps for improved and local varieties, respectively, with female plot managers realizing less than their male counterparts. Improved groundnut seeds increase female plot manager’s yields but not the yields of male plot managers. Male advantage and female disadvantage combined account for more than 70% of the yield gap in both improved and local groundnut variety production and exceed pure productivity differences. Labor use differences between female and male plot managers and variety types explain the observed yield gap. Interventions and policies that increase women’s access to productive inputs including improved seed will significantly contribute to closing the yield gap, and thereby increase crop production, food security, as well as women’s incomes.

## Introduction

The question of why male farmers across sub-Saharan Africa reportedly achieve higher productivity than their female counterparts has been the focus of a number of important studies in recent years (FAO, 2011) as it has direct effects on both food production as well as gender equity if the underlying causes are well understood (FAO, 2019, World Bank, FAO, IFAD, 2009). Researchers have sought to understand the ‘gender yield gap’ in various contexts by focusing on a range of variables that may account for lower crop yields in women’s plots. Studies have measured gender gap in agriculture productivity using different indicators ranging from the sex of the household head to the sex of plot manager and the sex of the resource controller (Peterman, Quisumbing, Behrman, & Nkonya, 2011; Quisumbing, Payongayong, Aidoo, & Otsuka, 2001). Peterman et al. (2011) found lower productivity on female-managed plots even when household-level unobservable factors were controlled for. Ali, Bowen, Deininger, and Duponchel (2016) reported that women-managed plots were on average 20%–30% less productive compared to plots managed by men. This increases male – female income inequality in agriculture and leads to an overall unattained potential given that women comprise 50% of the total labor in agriculture in Sub-Saharan Africa. Similarly, Croppenstedt, Goldstein, & Rosas, 2013 found that female farmers had a lower output per unit of land and were much less likely to be active in commercial farming than their male counterparts. The observed fact that female farmers operate at lower productivity levels than their male counterparts, has been attributed to gender differences in access to inputs, resources and services (Croppenstedt et al., 2013). Many of the gender studies on agricultural yield gaps argued that the lack of access to finance impedes female entrepreneurship and prevents women from participating in the modern market economy. Research in Southern and Eastern Africa has pointed out that women are more likely to be excluded from financial markets than men which hinders their productive capacities (Aterido, Beck, & Iacovone, 2013). Other authors have pointed to the constrained access to and control over key productive assets and resources such as land as the main causes for the widening gender yield gap (Wanjala, 2014). Other studies reported less active policies that would support women’s access to inputs and services such as fertilizers, credit, tenure security, market and extension services which affect plot management and marketing of agricultural produce (Horrell & Krishnan, 2007; Quisumbing et al., 2001; Tiruneh, Testfaye, Mwangi, & Verkuijl, 2001; Udry, Hoddinott, Alderman, & Haddad, 1995).

The observed fact is that female farmers tend to focus on food crop production which, in some regions, have been referred to as ‘women’s crops’ (Doss, 2002), one example being groundnuts in Uganda. Women often give priority to food crops to ensure the household is food secure. Additionally, male dominance in ownership and use-control of land leads to difficulties for female plot managers to grow the mostly perennial commercial crops (de la O Campos, Covarrubias, & Patron, 2016).

Based on the fact that the biggest share of workers in agriculture in Uganda are women (FAO, 2011), closing the gender yield gaps will unlock the agricultural potential of women in Uganda and thereby advance food security and sustainable human development. Thus, bridging the yield gap between male- and female-managed plots will contribute to the achievement of several Sustainable Development Goals especially the first (no poverty), second (zero hunger) and fifth (gender equality). Different studies investigating the gender yield gaps explore the use of different aggregation and focus levels of gender variables between plot and household level. At plot level, the variables used in most studies include the plot owner, plot manager or the plot holder (Goldstein & Udry, 2008; Peterman et al., 2011). Studies in Uganda and Malawi that explored the differences in productivity when plots are co-owned and co-managed by a man and a woman showed varying results. For instance, the study in Malawi found comparable gender gaps when using the overall sample and a sub-sample of plots co-managed by men and women. The study in Uganda found that mixed-plot ownership captured the impact of unobserved household characteristics when household-fixed effects are not controlled for (Kilic, Palacios-Lopez, & Goldstein, 2013; Peterman et al., 2011).

In this study, we focused on groundnut as a ‘woman’s crop’ (Orr, Tsusaka, Homann-KeeTui, & Msere, 2014) and the second most important staple legume in Uganda, to measure gender yield differences at plot level, with a hypothesis that yields from female-managed plots were lower than from male-managed plots. The objective was to determine the magnitude of the gender yield gap between male- and female-managed groundnut plots and explore the underlying causes of this yield gap. Understanding the yield gap determinants for one important staple crop in Uganda and one of the most common ‘women’s crop’ across Africa will enable a closer understanding of the root causes of gender disparities in groundnut production. This will allow development practitioners as well as policymakers to improve the design and delivery of project interventions. Ultimately, this will not only improve the functioning of the groundnut sector in Uganda but also increase the availability of this important crop while improving women’s participation and income earning opportunities.

## Data and methodology

### Data collection

This study was conducted in Northern and Eastern Uganda, the two leading groundnut producing regions (Okello, Biruma, & Deom, 2010; Okello, Deom, Puppala, Monyo, & Bravo-Ureta, 2018). We employed a mixed methods approach collecting gender disaggregated quantitative and qualitative data. For the quantitative part, groundnut plot managers were stratified by type of groundnuts grown (improved or local varieties). From each stratum, we proportionately randomly sampled women and men plot managers leading to 58% female respondents due to their high involvement in groundnut production. A plot manager was defined as the person who initiates the cultivation of a plot of land and uses his/her skills or hires skills and labor to produce groundnut and/or other crops on that plot. Primary data were collected using a pre-tested questionnaire from a total sample of 228 respondents of whom 58% were women. The data collected included socio-economic characteristics of the plot managers, types and varieties of groundnuts grown, farming practices used, land allocated to groundnuts and the respective output, and information about decision making in groundnut production. Additionally, we explored farmers’ knowledge of groundnut seeds and varieties grown, important varietal traits associated with the selected varieties, as well as seed/variety replacement decisions and behavior and impacts these had on yield outcomes by gender. The data were cleaned and analyzed using SPSS 20.0 (Armonk, NY, USA) and STATA14.0 (College Station, TX, USA).

The qualitative data were collected in two phases, one dataset was concurrently collected with the quantitative data (concurrent triangulation mixed method) in 12 Focus Group Discussions (FGDs) and the second was a follow up dataset (sequential explanatory mixed method) (Bryman, 2006; Creswell, Plano Clark, Gutmann, & Hanson, 2003). In both qualitative data collection phases, women were interviewed separately from men. A structured guide was used to elicit responses on key aspects of groundnut production, marketing and utilization at plot and household levels. The sequential explanatory phase involved data collection through a series of 48 FGDs using a designed vignette-based guide on the life of a married couple in the community that was presented to men and women plot managers. The FGD respondents were selected from the same villages included in the quantitative household survey.

### Data analysis

A combination of descriptive analysis and econometric methods were used. The gender yield gap was defined as the difference between the mean groundnut yields obtained from female- and male-managed plots. The Oaxaca-Blinder model was then used to investigate the variables that contribute to the gender differentials in groundnut production. First introduced by Oaxaca (1973) and Blinder (1973), and later by Cotton (1988) and Neumark (2004), the Oaxaca-Blinder model has been widely used in measuring gaps of various outcomes between groups. It has been applied to study wage gaps in Tunisia, Germany and the UK (Jeddi & Malouche, 2015; Machin & Puhani, 2003), wage differentials between urban and rural-urban migrant workers in China (Zhang, Sharpe, Li, & Darity, 2016) and wage and education differentials in Portugal (González, Santos, & Santos, 2009). Elder, Goddeeris, and Haider (2010) indicated that the Oaxaca-Blinder model can be used to estimate unexplained gaps in various mean outcomes.

Recently, scholars have increasingly used the Oaxaca-Blinder model to study the gender gaps in agricultural productivity (Aguilar, Carranza, Goldstein, Kilic, & Oseni, 2015; Ali et al., 2016; Backiny-Yetna & McGee, 2015; Oseni, Corral, Goldstein, & Winters, 2015). Many of these have been macro-level studies, with few such as Mukasa and Salami (2015) focusing on the micro-level to capture gender differentials in outcomes such as yields at farmer level. This study contributes to the micro-level gender differentials literature by analyzing the groundnut gender yield gap in Uganda at plot level.

We label the two farmer categories, women and men, as group W for female plot managers and group M for male plot managers. The mean yield difference to be explained ($\Delta \bar{Y}$) is the difference of the mean yields for the women group W and men group M, denoted as ${\bar{Y}}_{W}$ and ${\bar{Y}}_{M},$ respectively. Hence,

(1) $$\Delta \bar{Y_{}}={\bar{Y}}_{M}-{\bar{Y}}_{W}$$

Building from the context of a linear regression, the mean outcome for group G∈{M,W} can be expressed as ${\bar{Y}}_{G}={\bar{X}}_{G}^{\prime }{\overset{\wedge }{\beta }}_{G}$

where ${\bar{X}}_{G}$ contains the mean values of the explanatory variables and ${\overset{\wedge }{\beta }}_{G}$ are the estimated regression coefficients. Therefore, $\Delta \bar{Y_{}}$ is re-written as;

(2) $$\Delta \bar{Y_{}}={\bar{X}}_{M}^{\prime }{\overset{\wedge }{\beta }}_{M}-{\bar{X}}_{W}^{\prime }{\overset{\wedge }{\beta }}_{W}$$

The expression in Equation (2) can, in turn, be written as the sum of the following three terms: endowments, coefficients and interaction in that order.

(3) $$\Delta \bar{Y_{}}={\left({\bar{X}}_{M}-{\bar{X}}_{W}\right)}^{\prime }{\overset{\wedge }{\beta }}_{W}+{\bar{X}}_{W}^{\prime }\left(\overset{}{{\bar{\beta }}_{M}-{\overset{\wedge }{\beta }}_{W}}\right)+{\left({\bar{X}}_{M}-{\bar{X}}_{W}\right)}^{\prime }\left({\overset{\wedge }{\beta }}_{M}-{\overset{\wedge }{\beta }}_{W}\right)$$

Equation (3) represents the threefold Blinder-Oaxaca decomposition of the groundnuts yield difference between men- and women-managed plots. The first part on the right-hand side is the endowments term that represents the contribution of differences in explanatory variables across groups. These variables are presented in Table 1. The second part on the right-hand side is the coefficients term; it is the part that is due to group differences in the coefficients. The third part is the interaction term that accounts for the fact that cross-group differences in explanatory variables and coefficients can occur at the same time.

**Table 1.** Variables used in the Blinder-Oaxaca yield gap model.

Dependent variable = Farmer’s groundnut yield (Kg/ha)	
Explanatory variables	Variable Measurement/description
Pest and disease constraint (Yes = 1, No = 0)	Dummy (if farmer considers pests and diseases to be a serious groundnut production constraint)
Region (Northern = 1, Eastern = 0)	Regional location of the farmer in Uganda
Seed productivity trait index^a^	Index of six groundnut seed productivity attributes farmers consider when selecting seed
Whether the farmer practiced timely weeding (Yes = 1, No = 0)	Dummy (farmer weeded his/her groundnuts on time)
Number of family members involved in groundnut harvest	Number of household members who participated in the harvest of groundnuts in the last two seasons
Whether the farmer perceived soil infertility as a production constraint (Yes = 1, No = 0)	Dummy (farmer perception of his/her groundnut plot soil fertility)
Total land owned by household farmer (ha)	Total area of land owned by the groundnut farmer
How much credit a farmer obtained from VSLA (US$)	Amount of money farmer borrowed from a village savings scheme
Farmers’ age (years)	Age of the farmer
Farmer considers high yield seed at seed selection (Yes = 1, No = 0)	Dummy (Farmer considers the yield capacity of groundnut seed at seed selection)
Crop diversity index	Index calculated as number of crops grown by farmer out of total available in the area
Total annual seed and chemical costs (US$)	Annual seed and chemical costs spent on groundnuts
Total annual hired labor (man hours)	Total man hours of hired labor used in groundnuts annually
Farmer’s education level (years)	Level of education (number of years at formal school) of the farmer
Woman decides on how to spend income from groundnuts (Yes = 1, No = 0)	Dummy (woman has power to decide how to use the income earned from groundnuts)

a Seed productivity trait index calculated from binary responses on the farmer’s perception of; yielding capacity, early maturity, grain size, pods per plant, pod size, resistance to pests and diseases.

In addition, we estimated a two-fold Blinder-Oaxaca decomposition in which we decompose the mean groundnut yield outcome difference between men and women with respect to a vector of reference coefficients ${\overset{\wedge }{\beta }}_{R}$ as indicated in Equation (4);

(4) $$\Delta \bar{Y_{}}={\left({\bar{X}}_{M}-{\bar{X}}_{W}\right)}^{\prime }{\overset{\wedge }{\beta }}_{R}+{\bar{X}}_{M}^{\prime }\left(\overset{}{{\bar{\beta }}_{M}-{\overset{\wedge }{\beta }}_{R}}\right)+{\bar{X}}_{W}^{\prime }\left({\overset{\wedge }{\beta }}_{R}-{\overset{\wedge }{\beta }}_{W}\right)$$

The first part on the right-hand side gives the explained difference, the second part gives the unexplained difference for men (M) and the third part represents the unexplained difference for women (W). We also estimated a selection bias corrected Oaxaca-Blinder model using a Heckman link to address the limitations posed by selection bias within the data.

Among the key variables (Table 1) that were hypothesized to influence the groundnut gender yield gap, were the farmer’s field pest and disease burden, perceived soil fertility constraint, and seed productivity trait index as calculated from the attributes that farmers consider while selecting groundnut seed. This index measures the score a farmer attains on a six-point scale of six seed traits related to seed productivity considered at seed selection that was expected to influence yield positively as it tends to unity. We also calculated and introduced in the model the crop diversity index. We defined this index as the proportion of the number of crops a farmers grows on their farm out of the total number of crops available and grown in the community. A high crop diversity is expected to increase the gender yield gap since the more crops are grown, the more the labor and other limited capital resources are spread over a wider range of crops which in turn limits the attention to each individual crop and thus reducing the groundnut yields. The number of family members involved in groundnut harvest was also considered as harvesting is one of the most labor-intensive production activities. In addition, the timely weeding dummy as a proxy for sufficient access to labor was used in the model. We used a dummy variable to assess whether a woman’s contribution to the decision making on how to spend groundnut income, influenced her performance in groundnut production. This dummy was a proxy for control over the resource. The financial opportunities and investment levels were captured using the amount of credit a farmer obtained from a village savings and credit scheme (VSLA) and the total annual seed and chemical costs for groundnuts.

## Results and discussion

### Gender differences in groundnut production

Results in Table 2 indicate that women growing groundnuts, whether improved or local varieties realized lower yields than men. The gender yield gap was significantly higher (about 41%) when both women and men are growing local varieties, but reduced to only 14% when both were growing improved varieties. The results show that about 55% of women who grew either of the varieties managed to weed on time compared to 88% of men growing improved and 53% of men growing local varieties. Men growing improved varieties borrowed more and invested more in hired labor, seed and pest/disease control chemicals compared to women. Only 29% of the women growing improved varieties and 37% growing local varieties stated that they made decisions on how to spend the income earned from their groundnut sales. In the majority of cases, decisions were made by men (their spouses). This contradicts the common notion that groundnut is a woman’s crop and suggests rather that men are more likely to dominate even in this so called ‘women’s crops’ if they are destined to the market (Carr, 2008; Doss, 2002).

**Table 2.** Summary statistics of variables used in the Oaxaca-Blinder model.

	Pooled sample	Farmer grew improved varieties				Farmer grew local varieties			
** **	(*n* = 228)	Women (*n* = 41)		Men (*n* = 34)		Women (*n* = 92)		Men (*n* = 61)	
Variable	Mean (Std. Dev.)	Mean	Std. Dev.	Mean	Std. Dev.	Mean	Std. Dev.	Mean	Std. Dev.
Groundnut yield (Kg ha^−1^)	638.71^b^ (570.81)	499.27^b^	380.43	789.52	408.63	447.33	369.69	621.15	579.49
Pest and disease constraint (%)	16.22^b^	12.20	–	5.88	–	19.57	–	19.67	–
Region (North, %)	13.83^b^	19.51	–	14.71	–	47.83^b^	–	31.15	–
Seed productivity trait index	6.29	10.57	–	10.29	–	3.08	–	6.01	–
Whether the farmer practiced timely weeding (Yes = 1, No = 0)	59.65	56.10^c^	–	88.24	–	55.43	–	52.46	–
Number of family members involved in groundnuts harvest	5.00	5.00		5.00		6.00		4.00	
Total land owned by household farmer(ha)	4.62^c^ (2.83)	4.82	3.12	5.84	2.96	3.85^c^	2.61	4.98	2.59
How much credit a farmer obtained from VSLA (US$)	60.47 (66.99)	88.45	101.09	53.99	49.69	47.20	49.92	91.26	175.87
Farmer's age (years)	43.00^b^ (13.00)	39.76^b^	11.71	46.00	11.74	42.17	12.93	44.77	14.84
Farmer considers yield attribute at seed selection (%)	71.49	82.93	–	79.41	–	65.22	–	68.85	–
Crop diversification index (number of crops grown by farmer out of total available in the area)	0.27^b^ (0.15)	0.23	0.16	0.21	0.14	0.31	0.15	0.25	0.14
Total annual seed and chemical costs spent on groundnuts (US$)	45.22^b^ (64.72)	46.57	65.64	70.06	80.37	32.49^a^	56.46	51.07	64.04
Total annual hired labor (man hours)	618.91^a^ (5271.66)	152.13	807.98	2101.10	11,138.45	24.44	29.97	922.25	5596.93
Farmer’s education level (years in school)	6.45^c^ (3.55)	6.88^a^	3.03	8.06	2.94	4.96^c^	3.77	7.51	3.02
Woman decides on spending income groundnuts (%)	37.28	29.27	–	47.06	–	36.96	–	37.70	–

Significance (Women-men comparison): ^a^10%, ^b^5%, ^c^1%.

Results generally show no gender differences in labor used in groundnut production. However, female plot managers growing local groundnut varieties used significantly (*p* < .10) more family labor in harvesting than their male counterparts. We also found that female plot managers growing local varieties use slightly more labor in seedbed preparation than male plot managers (Table 3).

**Table 3.** Labor use by gender in groundnut production.

	Number of workers used annually per activity Mean (Std. Dev)					
	Local variety			Improved		
	Women	Men	Pooled sample	Women	Men	Pooled sample
Activity	Family labor					
Bush clearing	4.33 (2.51)	4.14 (3.12)	4.25 (2.76)	5.57 (6.04)	4.2 (2.84)	4.93 (4.82)
Seedbed preparation	8.63 (8.15)	7.15 (5.30)	8.04 (7.18)	6.16 (6.44)	6.86 (6.91)	6.47 (6.61)
Groundnut planting	5.56 (3.54)	5.7 (5.15)	5.62 (4.21)	5.36 (3.74)	6.21 (5.83)	5.744.76
Weeding	4.63 (2.54)	5.94 (3.80)	5.03 (3.01)	5.94 (4.42)	6.38 (7.46)	6.08 (5.45)
Groundnut harvesting	6.42^a^ (6.16)	4.41 (4.55)	5.65 (5.66)	6.22 (6.37)	5.17 (5.60)	5.76 (5.99)
** **	Hired labor					
Bush clearing	6.96 (9.52)	5.92 (12.06)	6.46 (10.72)	4.13 (2.87)	3.38 (2.22)	3.79 (2.58)
Seedbed preparation	8.21 (13.81)	10.98 (14.22)	9.41 (13.98)	7.59 (5.96)	7.92 (5.94)	7.75 (5.89)
Planting	6.64 (6.78)	8.14 (9.40)	7.43 (8.25)	5.4 (4.86)	6.83 (4.90)	6.18 (4.88)
Weeding	10.15 (9.80)	10.14 (8.12)	10.14 (9.04)	6.7^b^ (3.37)	10.91 (7.28)	8.48 (5.72)
Harvesting	7.07 (8.73)	6.75 (7.52)	6.92 (8.03)	4.11 (2.67)	3.13 (2.03)	3.65 (2.37)

Significance: ^a^10%, ^b^1%.

Generally, irrespective of gender and the groundnut varieties grown, farmers hired labor mainly for weeding, seedbed preparation and harvesting. However, male plot managers growing improved groundnut varieties used significantly more labor for weeding. Doss (2018) asserted that the usual measure of labor input – time spent working on a farm or plot – does not necessarily account for knowledge or management skills. A family member might work for few hours but provide knowledgeable direction to others.

However, Palacios-Lopez, Christiaensen, and Kilic (2015) estimated female labor share in crop production in Uganda at slightly above 50%. The authors, however, added that although there are no systematic differences across crops and activities, female labor shares tend to be higher in households where women own a larger share of the land and when they are more educated.

### Gender differences in groundnut yields

Table 4 indicates that without correcting for selection bias, the mean log yield in improved groundnut varieties is 6.2 and 6.7 for women and men, respectively, giving a geometric mean of 491 kg ha^−1^ for women and 775 kg ha^−1^ for men (a gap of 63%). For local varieties, the women plot managers realized a yield of 356 kg ha^−1^ while the men got 504 kg ha^−1^ (a gap of 44%). This confirms that, compared to men, women are disadvantaged in terms of yield whether they grow improved or local varieties. Some studies such as Mnimbo et al. (2017) have explored the linkage between gender roles and crop choices and found clear differences between women and men. Women were found to prioritize food crops over considerations of the commercial aspects of their farming or crops.

**Table 4.** Oaxaca uncorrected for selection bias pooled model for gender yield gap in groundnut production.

	Uncorrected for selection bias model					
	Farmer used improved seed			Farmer used local/indigenous seed		
Yield	Coefficient (Robust Std. Err)	[95% Conf. Interval]		Coefficient (Robust Std. Err)	[95% Conf. Interval]	
Differential						
Prediction_1 (Women)	6.20^a^ (0.28)	5.65	6.74	5.86^a^ (0.17)	5.55	6.21
Prediction_2 (Men)	6.65^a^ (0.23)	6.20	7.10	6.22^a^ (0.22)	5.80	6.65
Difference	−0.46 (0.36)	−1.17	0.25	−0.35 (0.28)	−0.89	0.19
Decomposition						
Explained	0.36 (0.34)	−0.32	1.03	0.05 (0.23)	−0.39	0.49
Unexplained	−0.81^b^ (0.33)	−1.46	−0.17	−0.40 (0.30)	−0.99	0.19
Endowments	3.46 (4.66)	−5.66	12.58	−0.36 (0.39)	−1.140	0.391
Coefficients	−1.61^b^ (0.72)	−3.03	−0.20	12.65 (17.36)	−21.38	46.68
Interaction	−2.30 (4.68)	−11.48	6.87	−12.62 (17.39)	−46.70	21.46

Significance: ^a^1 and ^b^5% level, respectively.

When correcting for selection bias, the yield achieved by women plot managers in both improved and local groundnut varieties did not change but mean log yields for men plot managers were adjusted upwards to 7.470 (geometric mean yield of 1755 kg ha^−1^) for improved, and downwards to 5.441 (geometric mean yield of 231 kg ha^−1^) for local varieties (Table 5). These results reveal that after correcting for selection bias, male plot managers perform even better.

**Table 5.** Oaxaca corrected for selection bias (Heckman) model for gender yield gap in groundnut production.

	Corrected for selection bias model					
	Farmer used improved seed			Farmer used local/indigenous seed		
Yield	Coefficient (Robust Std. Err)	[95% Conf. Interval]		Coefficient (Robust Std. Err)	[95% Conf. Interval]	
Differential(gap)						
Prediction_1 (Women)	6.20^a^ (0.28)	5.65	6.74	5.88 (0.17)	5.55	6.21
Prediction_2 (Men)	7.47^a^ (2.83)	1.93	13.01	6.11 (0.34)	5.44	6.78
Difference	−1.28 (2.84)	−6.84	4.29	−0.24 (0.38)	−0.98	0.51
Endowments	3.13 (6.31)	−9.23	15.49	−0.38 (0.29)	−0.94	0.18
Coefficients	−2.43 (2.91)	−8.14	3.28	12.76 (16.05)	−18.69	44.21
Interaction	−1.97 (6.33)	−14.37	10.43	−12.62 (6.05)	−44.07	18.83

^a^1% level.The selection model is estimated with household size, expected groundnut yield, area allocated to groundnuts annually and woman having power to decide on use of land as selection variables.

Table 5 indicates that if women plot managers had the same endowment (asset and resource base level) as men, their yields would increase by 49.5% for improved varieties and would slightly reduce by 6% for local varieties. If the male plot managers’ coefficients were applied to female plot managers, their yields would reduce by 39% for improved varieties but increase by 217% for local varieties which suggests that women prioritize local varieties. This also indicates that, in the current situation, women have a comparative disadvantage in growing the more productive improved varieties compared to the less productive local varieties which emanates from their gender positioning in society that limits their production and marketing opportunities. The interaction term that measures the simultaneous effect of differences in endowments and coefficients between women and men indicates a reduction in female yields for both improved (32%) and local varieties (215%). Therefore, both endowment and coefficient differences work in the same direction and increase gender yield gaps.

### Determinants of gender yield gap in groundnut production

Most economic theories consider the household as a unit of analysis and pay little attention to intra-household differences in resource allocation. However, in many cases, agricultural production occurs on multiple plots which are each controlled by different household members including women and men. These theories also point to the fact that household members consider risks in their decision making (Humphrey & Verschoor, 2004). Ultimately, farmers choose to allocate resources across multiple plots and household members which in turn results in the observed yields (Galarza, 2009; Harrison, Humphrey, & Verschoor, 2010).

With this in mind, our results show that the seed productivity trait index had a significantly (*p* ≤ .10) positive influence on yields of the female-managed plots of improved groundnut varieties, while this does not show for local varieties. In conformity with this finding, Asrat, Yesuf, Carlsson, and Wale (2010) found that farmers value yield when selecting varieties and in their willingness to pay for seed.

With more land owned, yields of female-managed plots with improved groundnut varieties reduced significantly (*p* ≤ .01) while yields of male-managed plots with local varieties increased significantly (*p* ≤ .10). For male plot managers, however, growing local varieties on more land is indicative of more commercial production as they intensify their production with expanding areas and do not face the same constraints as their female counterparts. This resource-access based result is corroborated by the finding of Udry (1996) that the marginal productivity of land controlled by women is lower than that of land controlled by their husbands. Peterman et al. (2011) also reported lower agricultural productivity on female owned plots in Uganda and Nigeria. Mukasa and Salami (2015) found that female-managed plots in Uganda were about 31% less productive than male-managed plots. This was higher than in Tanzania and Nigeria where the gap was estimated at 19% and 27%, respectively. Generally, female-managed farms have been found to have low productivity due to their smaller size and resource constraints.

Timely weeding of groundnuts was found to have a positive influence on yields of female-managed plots for both improved and local varieties. This is founded in the availability and allocation of labor to groundnuts and other crops and further reflected in the positive sign of the variable controlling for total hired labor for women. This result resonates well with the findings of O'Laughlin (2007) and Udry (1996) that women used more family labor on own plots than men and hired less labor.

Results further show that high crop diversification for women growing improved groundnut varieties had a significant (*p* ≤ .01) and positive influence on yield (Table 3). A marginal increase in women’s diversification index by one would increase their groundnut yields by about six times. This can be explained by the fact that with more diversification, women can spread their production and marketing risks allowing them to use the income from other crops to invest in improved inputs, technologies and practices. This can have significant knock-on effects as Okello et al. (2015) reported that improved groundnut varieties had significant higher profit margins and lower break-even yields than the local varieties.

### Underlying causes of observed yield gap between women and men

From the Focus Group discussions (FGDs), we found gender differences in groundnut trait preferences. For instance, ease of cooking, oil content, (sweet) taste, ease of plucking are the traits highly sought after by female groundnut producers. We also found that the women shared their seeds amongst themselves more often than men.

From the concurrent triangulation of qualitative data, we found that women in Northern Uganda were mainly constrained by access to and control over land. The clan elders allocate the land and women cannot inherit land permanently. When a woman feels like selling off land for reasons such as the desire to get more productive land elsewhere, she has to sell to a clan member. Such cultural biases are likely to contribute to the widening of the gender yield gap. Results in Table 6 show that adjusting women’s endowments to men’s levels would increase their current yields by 43% in improved varieties and 5.1% in local varieties. These results indicate that a combination of improved technologies and increased access to productive assets for women could help to increase women’s competitiveness compared to men and thereby contribute to poverty reduction. This conclusion is supported by Mehra and Rojas (2008) who found that higher crop yields contribute to poverty reduction even if the gap cannot be fully closed. However, the results leave an unexplained gap of 44.4% in improved varieties and 67.2% in local varieties which could be based on exogenous or power and control factors. The women in FGDs indicated having less control over groundnuts for sale but more control over groundnuts for home consumption. However, they concurred that many of their spouses give them money from groundnut sales, though they may have limited power to decide on what to spend it (Table 7).

**Table 6.** Decomposition model by farmer gender and variety type produced.

	Farmer used improved seed variety		Farmer used local seed variety	
	Women	Men	Women	Men
Variable	Coef. (Std. err)	Coef. (Std. err)	Coef. (Std. err)	Coef. (Std. err)
Pest and disease constraint (Dummy)	1.00 (1.20)	0.80 (1.32)	0.09 (0.40)	0.98 (0.62)
Region (North = 1, East = 0)	−2.34^c^ (0.92)	−2.50 (3.30)	−0.17 (0.56)	0.27 (0.62)
Seed productivity trait index	2.79^c^ (1.10)	1.56 (2.41)	−1.33 (2.57)	0.48 (1.42)
Whether the farmer practiced timely weeding (Yes = 1, No = 0)	1.43^c^ (0.63)	0.96 (0.81)	0.76^b^ (0.33)	−0.34 (0.45)
Total land owned by household farmer (ha)	−0.26^a^ (0.05)	0.08 (0.10)	0.00 (0.08)	0.20^c^ (0.10)
How much credit a farmer obtained from VSLA (US$)	−0.01 (0.00)	−0.00 (0.01)	−0.01^a^ (0.00)	−0.00 (0.00)
Farmer considers high yield seed at seed selection (dummy)	−1.50^c^ (0.62)	0.70 (0.61)	0.42 (0.39)	0.28 (0.80)
Crop diversification index (number of crops grown by farmer out of total available in the area)	5.81^a^ (1.17)	7.44 (9.88)	−1.51 (1.48)	0.09 (1.99)
Total annual seed and chemical costs spent on groundnuts (US$)	92.65 (202.51)	−106.83 (344.02)	−15.84 (16.70)	368.91 (236.35)
Total annual hired labor (man hours)	0.00 (0.00)	0.01 (0.01)	0.01 (0.01)	−0.00^a^ (0.00)
Farmer’s education level (years in school)	−0.14 (0.09)	−0.03 (0.21)	−0.02 (0.08)	0.04 (0.09)
Woman decides on how to spend income from groundnuts (dummy)	−1.25 (0.71)	0.60 (1.28)	0.52 (0.32)	−1.11^b^ (0.49)
Constant	7.02^a^ (0.90)	3.23 (2.40)	6.01^a^ (0.88)	3.46^b^ (1.24)
Model summary				
Number of observations	38.00	29.00	69.00	52.00
Prob >*F*	0.00	0.04	0.00	0.00
*R*^2^	0.95	0.77	0.41	0.65

Significance: ^a^1%, ^b^5% and ^c^10% levels, respectively.

**Table 7.** Geometric means decomposition of yield gap by gender and variety type.

Yield	Improved variety			Local/indigenous variety		
	exp(b) (kg ha^−1^)	[95% Conf. Interval]		exp(b) (kg ha^−1^)	[95% Conf. Interval]	
Differential(gap)						
Prediction_1(women)	490.61	296.10	812.91	356.18	268.25	472.92
Prediction_2(men)	774.76	567.81	1057.16	504.38	348.24	730.54
Difference	63.30%	35.00%	114.60%	70.60%	44.30%	112.60%
Decomposition						
Explained	1.43	0.73	2.801	1.051	0.676	1.635
Unexplained	44%	23%	84.50%	67.20%	37.20%	121.30%

Results further indicate that the differences in characteristics such as access to land, education and credit contributed 49% of the yield differential in improved groundnut varieties and 71% in local varieties for men plot managers as compared with 22% for the women plot managers growing improved groundnut varieties and 92% for women growing local varieties. This result is in agreement with a study by Hegarty and Pratto (2001) that noted that group differences can be explained by social norms. The selected variable parameters contributed to similar proportions for women and men growing both improved and local varieties (Table 8). This decomposition enables segregation of the gaps into the percentage contribution by characteristics and the selected variable parameters (Sinning, Hahn, & Bauer, 2008).

**Table 8.** Decomposition of the yield differential between men and women using a linear regression model.

	Improved variety		Local variety	
Results	Coef.	Percentage	Coef.	Percentage
Omega = 1 (Men)				
Characteristics	−3.46	−757.49%	0.38	107.71%
Coefficients	3.92	857.49%	−0.03	−7.71%
Omega = 0 (Women)				
Characteristics	−1.157	−253.22%	13.00	3,735.92%
Coefficients	1.614	353.22%	−12.65	−3,635.92%
Omega = wgt (Neumark weight)				
Productivity	0.10	22.80%	0.10	28.77%
Advantage	0.18	39.64%	0.14	40.26%
Disadvantage	0.17	37.56%	0.11	30.97%
Raw	0.46	100%	0.35	100%

Using the Neumark Weight, results indicated that differences in productivity between male and female plot managers contributed 23% to the yield gap in improved varieties and 29% in local varieties. The productivity advantage of men over women contributed 40% in both improved and local varieties while the disadvantage of women over men contributed 38% to the yield gap in improved varieties as compared to 31% in local varieties. These findings point to the fact that gender yield gaps are to a large degree a result of unexplained gender inequalities in the communities’ structural arrangement, that could be attributed to social norms, practices and beliefs, rather than a difference in technical ability to produce.

FDGs, for instance, indicated that one of the most common cultural practices was the gifting of seeds, mostly local varieties with the most preferred traits, to newly married couples by their parents and the community. Among the groundnut traits women mentioned are richness in oil content which is sometimes extracted to be used for smearing newborn babies as it is believed to prevent body rashes, butter quality and sweet taste which is important for confectionery use and sauce quality. In many cases, the newly married women would start their marriage journey with local seed varieties which are mostly less productive but with preferred traits putting them at a productivity disadvantage right from the start.

## Conclusions

The aim of this research was to estimate the gender yield gap in groundnut production in Northern and Eastern Uganda and establish the factors that influence the gap. The study found that the gender yield gap is significant whether plot managers grew improved or local groundnut varieties. However, the gap reduces when both male and female plots managers grew improved varieties. We find large disparities in endowments, plots manager characteristics and interaction effects between men and women plot managers indicating structural disadvantages of the latter.

While productivity differences do explain parts of the gender yield gap, gender advantage and disadvantage differentials combined account for an even higher proportion of the total observed yield gap. This result is indicative of the fact that changing gender relations towards a more equitable sharing of resources and inclusion in decision making are also important drivers for closing the observed gender yield gaps. Empowering women in selecting varieties with high productivity traits, good agricultural practices, practicing crop diversification, timely farm operations, accessing key resources and ultimately deciding on where to invest the proceeds of their work will significantly reduce the yield gap. Understanding the traits that women value in groundnuts and the cultural mechanisms of exchanging local varieties would open a window for knowing what traits to pursue in improving groundnut varieties. In the case here, popularization of improved groundnut varieties and related technologies could be a good entry point as these improved varieties have already proven to close the yield gap and enable women to invest further into production and thereby improve their livelihoods. Overall, only removing structural barriers would allow female farmers to unlock their potential. A further detailed analysis of those structural barriers would be required to deepen the understanding of their root causes. Based on our results, we would argue that gender focused interventions and policies aimed at increasing women’s access to productive inputs, especially improved seed, can already contribute to reducing the yield gap.

## References

[CIT0001] Aguilar A., Carranza E., Goldstein M., Kilic T., & Oseni G (2015). Decomposition of gender differentials in agricultural productivity in Ethiopia. *Agricultural Economics*, 46, 311–334. doi:10.1111/agec.12167

[CIT0002] Ali D., Bowen D., Deininger K., & Duponchel M (2016). Investigating the gender gap in agricultural productivity: Evidence from Uganda. *World Development*, 87, 152–170. doi:10.1016/j.worlddev.2016.06.006

[CIT0004] Asrat S., Yesuf M., Carlsson F., & Wale E (2010). Farmers' preferences for crop variety traits: Lessons for on-farm conservation and technology adoption. *Ecological Economics*, 69, 2394–2401. doi:10.1016/j.ecolecon.2010.07.006

[CIT0005] Aterido R., Beck T., & Iacovone L (2013). Access to finance in Sub-Saharan Africa: Is there a gender gap? *World Development*, 47, 102–120. doi:10.1016/j.worlddev.2013.02.013

[CIT0006] Backiny-Yetna P., & McGee K (2015). *Gender differentials and agricultural productivity in Niger*. Washington, D.C: The World Bank.

[CIT0007] Blinder A. S (1973). Wage discrimination: Reduced form and structural estimates. *Journal of Human Resources*, 8, 436–455. doi:10.2307/144855

[CIT0008] Bryman A (2006). Integrating quantitative and qualitative research: How is it done? *Qualitative Research*, 6, 97–113. doi:10.1177/1468794106058877

[CIT0009] Carr E. R (2008). Men’s crops and women’s crops: The importance of gender to the understanding of agricultural and development outcomes in Ghana’s central region. *World Development*, 36, 900–915. doi:10.1016/j.worlddev.2007.05.009

[CIT0010] Cotton J (1988). On the decomposition of wage differentials. *The Review of Economics and Statistics*, 70, 236–243. doi:10.2307/1928307

[CIT0011] Creswell J. W., Plano Clark V. L., Gutmann M. L., & Hanson W. E (2003). Advanced mixed methods research designs In Tashakkori A. & Teddlie C. (Eds.), *Handbook of mixed methods in social and behavioral research* (pp. 209–240). Thousand Oaks: Sage.

[CIT0012] Croppenstedt A., Goldstein M., & Rosas N (2013). Gender and agriculture: Inefficiencies, segregation, and low productivity traps. *World Bank Research Observer*, 28, 79–109. doi:10.1093/wbro/lks024

[CIT0013] de la O Campos A. P., Covarrubias K. A., & Patron A. P (2016). How does the choice of the gender indicator affect the analysis of gender differences in agricultural productivity? Evidence from Uganda. *World Development*, 77, 17–33. doi:10.1016/j.worlddev.2015.08.008

[CIT0014] Doss C. R (2002). Men’s crops? Women’s crops? The gender patterns of cropping in Ghana. *World Development*, 30, 1987–2000. doi:10.1016/S0305-750X(02)00109-2

[CIT0015] Doss C. R (2018). Women and agricultural productivity: Reframing the Issues. *Development Policy Review*, 36, 35–50. doi:10.1111/dpr.12243 29263585 PMC5726380

[CIT0017] Elder T. E., Goddeeris J. H., & Haider S. J (2010). Unexplained gaps and Oaxaca–Blinder decompositions. *Labour Economics*, 17, 284–290. doi:10.1016/j.labeco.2009.11.002

[CIT0018] FAO (2011). *The state of food and agriculture 2010–2011: Women in agriculture: Closing the gender gap for development. Food and Agriculture Organization of the United Nations*. Rome, Italy: FAO.

[CIT0019] FAO (2019). FAO, gender equality and the 2030 Agenda. Retrieved from: http://www.fao.org/gender/background/en/

[CIT0020] Galarza F (2009). *Choices under Risk in Rural Peru (No. 17708)*. Germany: University Library of Munich.

[CIT0021] Goldstein M., & Udry C (2008). The profits of power: Land rights and agricultural investment in Ghana. *Journal of Political Economy*, 116, 981–1022. doi:10.1086/595561

[CIT0022] González P., Santos L. D., & Santos M. C (2009). Education and gender wage differentials in Portugal: What can we learn from an age cohort analysis? *Education Economics*, 17, 263–278. doi:10.1080/09645290802628437

[CIT0023] Harrison G. W., Humphrey S. J., & Verschoor A (2010). Choice under uncertainty: Evidence from Ethiopia, India and Uganda. *The Economic Journal*, 120, 80–104. doi:10.1111/j.1468-0297.2009.02303.x

[CIT0024] Hegarty P., & Pratto F (2001). The effects of social category norms and stereotypes on explanations for intergroup differences. *Journal of Personality and Social Psychology*, 80, 723. doi:10.1037//0022-3514.80.5.723 11374745

[CIT0025] Horrell S., & Krishnan P (2007). Poverty and productivity in female headed households in Zimbabwe. *Journal of Development Studies*, 43, 1351–1380. doi:10.1080/00220380701611477

[CIT0026] Humphrey S. J., & Verschoor A (2004). Decision-making under risk among small farmers in East Uganda. *Journal of African Economies*, 13, 44–101. doi:10.1093/jae/13.1.44

[CIT0027] Jeddi H., & Malouche D (2015). Wage gap between men and women in Tunisia. *arXiv Preprint arXiv*. Retrieved from https://arxiv.org/pdf/1511.02229.pdf

[CIT0028] Kilic T., Palacios-Lopez A., & Goldstein M (2013). *Caught in a productivity trap: A distributional perspective on gender differences in Malawian agriculture. Policy research working paper 6381*. Washington, DC: The World Bank.

[CIT0029] Machin S., & Puhani P. A (2003). Subject of degree and the gender wage differential: Evidence from the UK and Germany. *Economics Letters*, 79, 393–400. doi:10.1016/S0165-1765(03)00027-2

[CIT0030] Mehra R., & Rojas M. H (2008). *Women, food security and agriculture in a global marketplace. International Center for Research on Women (ICRW)*. Washington, D.C: ICRW

[CIT0031] Mnimbo T. S., Lyimo-Macha J., Urassa J. K., Mahoo H. F., Tumbo S. D., & Graef F (2017). Influence of gender on roles, choices of crop types and value chain upgrading strategies in semi-arid and sub-humid Tanzania. *Food Security*, 9, 1173–1187. doi:10.1007/s12571-017-0682-2

[CIT0032] Mukasa A. N., & Salami A. O (2015). Gender productivity differentials among smallholder farmers in Africa: A cross-country comparison. African Development Bank Group Working Paper, 231. Abidjan, Côte d’Ivoire. Retrieved from http://www.afdb.org/fileadmin/uploads/afdb/Documents/Publications/WPS_No_231_Gender_productivity_differentials_among_smallholder_farmers_in_Africa__A_cross-country_comparison.pdf

[CIT0033] Neumark D (Ed.). (2004). Employers’ discriminatory behavior and the estimation of wage discrimination In *Sex Differences in Labor Markets* (pp. 163–177). Abingdon, United Kingdom: Routledge.

[CIT0034] Oaxaca R (1973). Male-female wage differentials in urban labor markets. *International Economic Review*, 14, 693–709. doi:10.2307/2525981

[CIT0035] Okello D. K., Biruma M., & Deom C. M (2010). Overview of groundnuts research in Uganda: Past, present, and future. *African Journal of Biotechnology*, 9, 6448–6459.

[CIT0036] Okello D. K., Okori P., Puppala N., Boris B. U., Deom C. M., Ininda J., … Asekenye C (2015). Groundnut Seed Production Manual for Uganda. National Agricultural Research Organisation Entebbe, Uganda, January 2015. Retrieved from http://pmil.caes.uga.edu/documents/UGA203/Publication_UGA203_Deom_FY2015_GroundnutSeedProductionManualforUganda-Okello.pdf

[CIT0037] Okello D. K., Deom C. M., Puppala N., Monyo E., & Bravo-Ureta B (2018). Registration of ‘Serenut 6T’Groundnut. *Journal of Plant Registrations*, 12, 43–47. doi:10.3198/jpr2017.03.0016crc

[CIT0038] O'Laughlin B (2007). A bigger piece of a very small pie: Intrahousehold resource allocation and poverty reduction in Africa. *Development and Change*, 38, 21–44. doi:10.1111/j.1467-7660.2007.00401.x

[CIT0039] Orr A., Tsusaka T. W., Homann-KeeTui S., & Msere H. W (2014). What do we mean by ‘Women’s Crops’? A Mixed Methods Approach (No. 23). ICRISAT SocioEconomics Discussion Paper Series. Retrieved from http://oar.icrisat.org/8331/

[CIT0040] Oseni G., Corral P., Goldstein M., & Winters P (2015). Explaining gender differentials in agricultural production in Nigeria. *Agricultural Economics*, 46, 285–310. doi:10.1111/agec.12166

[CIT0041] Palacios-Lopez A., Christiaensen L., & Kilic T (2015). How much of the labor in African agriculture is provided by women? *Food Policy*, 67, 52–63. 10.1016/j.foodpol.2016.09.017 PMC5384444 28413246

[CIT0042] Peterman A., Quisumbing A., Behrman J., & Nkonya E (2011). Understanding the complexities surrounding gender differences in agricultural productivity in Nigeria and Uganda. *Journal of Development Studies*, 47, 1482–1509. doi:10.1080/00220388.2010.536222

[CIT0043] Quisumbing A., Payongayong E., Aidoo J. B., & Otsuka K (2001). Women’s land rights in the transition to individualized ownership: Implications for the management of tree resources in western Ghana. *Economic Development and Cultural Change*, 50, 157–182. doi:10.1086/340011

[CIT0044] Sinning M., Hahn M., & Bauer T. K (2008). The Blinder-Oaxaca decomposition for Nonlinear regression models. *The Stata Journal: Promoting Communications on Statistics and Stata*, 8, 480–492. doi:10.1177/1536867X0800800402

[CIT0045] Tiruneh A., Testfaye T., Mwangi W., & Verkuijl H (2001). *Gender differentials in agricultural production and decision-making among smallholders in Ada, Lume, and Gimbichu woredas of the central highlands of Ethiopia*. Mexico: International Maize and Wheat Improvement Center and Ethiopian Agricultural Research Organization.

[CIT0046] Udry C (1996). Gender, agricultural production, and the theory of the household. *Journal of Political Economy*, 104, 1010–1046. doi:10.1086/262050

[CIT0047] Udry C., Hoddinott J., Alderman H., & Haddad L (1995). Gender differentials in farm productivity: Implications for household efficiency and agricultural policy. *Food Policy*, 20, 407–423. doi:10.1016/0306-9192(95)00035-D

[CIT0048] Wanjala B. M (2014). Gendered Asset Inequalities in Africa. *Development*, 57, 472–480. doi:10.1057/dev.2015.26

[CIT0049] World Bank, FAO, IFAD (2009).The gender and agriculture sourcebook. Module 12 – Gender in crop agriculture. World Bank. Retrieved from http://siteresources.worldbank.org/INTGENAGRLIVSOUBOOK/Resources/CompleteBook.pdf

[CIT0050] Zhang L., Sharpe R. V., Li S., & Darity W. A (2016). Wage differentials between urban and rural-urban migrant workers in China. *China Economic Review*, 41, 222–233. doi:10.1016/j.chieco.2016.10.004

